# Nicotinamide and Pyruvate as Potential Therapeutic Interventions for Metabolic Dysfunction in Primary Open-Angle Glaucoma—A Narrative Review

**DOI:** 10.3390/jcm14227954

**Published:** 2025-11-10

**Authors:** Nathan Schanzer, Alon Harris, Kunal Kanwar, Rick Mortensen, Alice Verticchio Vercellin, Francesco Oddone, Carmela Carnevale, Keren Wood, Brent Siesky

**Affiliations:** 1 Department of Ophthalmology, Icahn School of Medicine at Mount Sinai, New York, NY 10029, USA; nathan.schanzer@mssm.edu (N.S.); rmortenson7@gmail.com (R.M.); alice.verticchio@mssm.edu (A.V.V.); keren.woodshalem@mssm.edu (K.W.); brent.siesky@mssm.edu (B.S.); 2 Feinberg School of Medicine, Northwestern University, Chicago, IL 60611, USA; kunal.kanwar@northwestern.edu; 3 Istituto di Ricovero e Cura a Carattere Scientifico: Fondazione G.B. Bietti per lo Studio e la Ricerca in Oftalmologia ONLUS, 00184 Roma, Italy; francesco.oddone@fondazionebietti.it (F.O.); carmela.carnevale@fondazionebietti.it (C.C.)

**Keywords:** glaucoma, pyruvate, mitochondria, nicotinamide, metabolism

## Abstract

Mitochondrial dysfunction and oxidative stress have been suggested as potential contributors to the initiation and progression of primary open-angle glaucoma (POAG). Nicotinamide and pyruvate are important in the human body for maintaining metabolic function and preserving cytoskeletal structures. Both substances show an age-dependent decline in humans which may contribute to metabolic dysfunction and POAG vulnerability. Pilot works suggest their consumption may help prevent retinal ganglion cell deterioration under elevated intraocular pressure (IOP) and oxidative stress. Currently, there are no approved POAG treatments to mitigate risks from non-IOP drivers of disease, including oxidative stress. The purpose of this review is to summarize and critically evaluate interventional studies that have investigated nicotinamide and pyruvate supplementation in attempts to treat metabolic dysfunction in POAG patients. A review of the relevant literature from October 1979 to November 2025 was performed using related search terminologies through PubMed, ClinicalTrials.gov, and Google Scholar, and by reference cross-matching of all related articles. Current pilot data suggests that supplementation with nicotinamide and pyruvate demonstrates certain aspects of retinal neuroprotection and produces short-term improvements in visual function. However, much of the existing work has been conducted in animal models, and human study data are severely limited in scope and duration. Several clinical trials are registered as being in progress that aim to determine the chronic effects of nicotinamide and pyruvate in humans. Long-term longitudinal investigations with significantly larger and diverse sample sizes tied to functional and structural outcomes are needed for the safety and potential clinical utility of nicotinamide and pyruvate for POAG.

## 1. Introduction

Glaucoma is a leading cause of irreversible blindness worldwide that is frequently associated with elevated intraocular pressure (IOP) [1]. The current medical management of primary open-angle glaucoma (POAG) is constrained to the singular concept of reducing IOP. Despite well-controlled IOP, a significant number of individuals with POAG will experience disease progression [2,3]. This highlights the urgent need for novel therapeutics that target the non-IOP drivers of POAG disease.

Mitochondria are organelles that are vital to energy generation, maintaining the balance of reactive oxygen species (ROS), and various processes involved in cellular homeostasis [4]. Mitochondrial abnormalities have been shown to occur prior to identifiable markers of neurodegeneration in aged mice [4]. Mitochondrial dysfunction has also previously been linked to the loss of retinal ganglion cells (RGCs) in glaucoma [5]. The human retina and optic nerve head (ONH) are rich in mitochondria and evidence from pilot studies suggests that mitochondrial dysfunction and elevated ROS are present in eyes with POAG [5,6,7,8,9,10,11], although not all studies agree [12].

Mechanistically, mitochondrial dysfunction can result in generation of ROS within retinal tissues, which may damage RGCs, including their DNA, proteins, and lipids [13,14]. Alterations in mitochondrial DNA (mtDNA) have been associated with POAG, with one study finding that 50% of POAG patients had a pathogenic mtDNA mutation [15]. Age-related mitochondrial dysfunction may also contribute to glaucomatous injury due to decreased repair capacity and increased apoptosis [5]. Given the role of mitochondrial metabolism in RGC survival, metabolic interventions represent a prime non-IOP target for the development of novel POAG therapeutics.

Targeting mitochondrial dysfunction as a neuroprotective strategy for patients with POAG has been previously discussed through the potential use of nicotinamide (NAM) and pyruvate [16,17,18,19]. NAM, nicotinamide riboside (NR), and niacin (vitamin B3 or nicotinic acid) are precursors to nicotinamide adenine dinucleotide (NAD). NAD acts as an oxidizing agent (NAD+) and a reducing agent (NADH) in cellular reactions, including oxidative phosphorylation and ATP production [18,20]. NAD acts as a substrate for enzymes maintaining mitochondrial homeostasis, Wallerian degeneration, and neuronal energy stores [21]. Pyruvate also plays an important role in the proper function of RGCs and their response to oxidative stress due to age, IOP, and ROS. Decreases in pyruvate function have wide-ranging impacts on energy metabolism, oxidative stress, and neuroinflammation, all of which have been associated with glaucoma [22,23]. Pyruvate function has been specifically shown to be disturbed during elevated IOP [22], and pyruvate depletion can render RGCs susceptible to poly(ADP-ribose) (PAR) polymerase-1 (PARP1)-mediated apoptosis [24].

In humans, NAM and pyruvate show an age-dependent decline, which may contribute to mitochondrial dysfunction, metabolic dysfunction, and ultimately, an increased vulnerability to POAG disease. Specifically, there is an age-related increase in NAD consumption and diminished NAD availability, leading to widespread oxidative dysregulation and susceptibility to damage from elevated IOP or decreased blood flow [18,25]. POAG is also known to increase with age [1].

As the goal of POAG management advances beyond IOP reduction, targeting mitochondrial dysfunction has emerged as a prime candidate for targeting metabolic intervention. NAM and pyruvate consumption have demonstrated various RGC neuroprotection properties, providing some justification for their potential role in POAG care. This review summarizes and discusses the various pilot interventional studies that have investigated NAM and pyruvate supplementation in humans, in an attempt to treat metabolic dysfunction in POAG. Furthermore, this review builds on the recent work of Kuang et al. [16], which summarizes novel therapeutic avenues for treatment of POAG, and D’Angelo et al. [26], which reviews ONH neuroprotection strategies in glaucoma. These articles provide a wide-ranging overview of mitochondria-targeted therapeutic strategies and neuroprotective compounds and go well beyond the scope of NAM and pyruvate (including vitamins and supplements such as Vitamin A, Vitamin C, ginkgo biloba, and many more). We sought to build upon this foundation and write a meticulous review, focused specifically on the compounds we believe to be especially promising, including NAM and pyruvate. Furthermore, we provide an update on the new literature that has come out since the previous review articles have been published. In this review, we strive to present an in-depth discussion of all of the relevant literature and ongoing clinical trials, using NAM and pyruvate for treatment of oxidative stress and metabolic dysregulation in POAG. We also provide deep insight into the future directions of research, specifically with regard to NAM and pyruvate.

## 2. Materials and Methods

A narrative review of the literature discussing NAM and pyruvate as interventions for POAG was conducted from 1 October 1979, to 1 November 2025, using PubMed (Bethesda, MA, USA), Google Scholar (Mountain View, CA, USA), and ClinicalTrials.gov (Bethesda, MA, USA). Keywords utilized in varying combinations include “glaucoma”, “mitochondria”, “nicotinamide”, “pyruvate”, “primary open-angle glaucoma”, “NAD”, “NAM”, “risk factors”, “POAG intervention”, “POAG treatment”, “oxidative stress”, “oxidative dysregulation”, “mitochondrial dysfunction”, and “mitochondrial stress”. The same keywords were used in all searches, and data were collected and organized using Microsoft Word (Microsoft Corporation, Redmond, WA, USA). Articles were screened for relevance and analyzed for inclusion in the paper. Inclusion criteria comprised interventional studies and ongoing clinical trials in animal or human models that used an NAD precursor supplement (i.e., NR, NAM, niacin), pyruvate, or both as interventional treatment (either prophylactic or remedial) in POAG. Studies or clinical trials were excluded if other antioxidant supplements, such as coenzyme Q10, citicoline, oleic acid, N-acetylcysteine, etc., were used alongside an NAD precursor or pyruvate.

A review of the literature yielded 106 potential articles of interest, of which 17 met the inclusion criteria and were not duplicate. We included interventional studies with both prospective and retrospective study designs in human subjects. We excluded any articles examining the effects of NAM or pyruvate in cellular models. The articles were screened by two independent reviewers (N.S. and K.K.), who screened each article by determining whether the article utilized either NAM, pyruvate, or both as an intervention in either a human or animal model. The two reviewers convened to discuss the articles they identified as eligible to include and resolved any disagreement by re-screening the article in question together, using the inclusion and exclusion criteria as a checklist to screen the article. The review of ongoing clinical trials yielded 12 trials, of which 8 were deemed relevant, actively recruiting subjects, and collecting data (according to clinicaltrials.gov). Clinical trials were included if they were using NAM, pyruvate, or both as an intervention. We excluded any trials from the discussion if they were using combination solutions, in which it would be difficult to determine whether the neuroprotective effect was coming from NAM or pyruvate independently or from the other compounds in the solution, such as coenzyme Q10 or N-acetylcysteine. These studies and trials were identified and included in the review, along with relevant references providing background information.

## 3. Results

### 3.1. Nicotinamide Supplementation in Animal Models of Glaucoma

Animal models involving NAM and glaucoma are highly varied and involve a variety of unique endpoints. The animal studies can be segmented into three main categories of research: (1). assessment of the genetic and biochemical basis of NAM supplementation; (2). NAM supplementation effects on mitigating the structural decay of RGCs and visual function; (3). studies examining the effects of NAM supplementation on cytoskeletal structure, including axon integrity and microtubule function. The animal models used in these studies comprise different strains of mice and rats. One of the advantages of using mouse and rat models in glaucoma research is that there is a high degree of conservation between mouse and human genomes. This enables genetic manipulation by altering the mouse or rat genome and the ability to breed the animal as desired. Furthermore, mouse and rat models are inexpensive and easy to handle, their eyes are easy to obtain, and the sample number for studies can be relatively large. Compared to other non-primate models, mice and rats share many characteristics of the anterior chamber, including the aqueous outflow pathway. Thus, results obtained from a rat model of glaucoma can be reasonably extrapolated to humans. The IOP elevation and retinal and ONH changes in glaucoma are also similar to those seen in humans, making these animals very good analogs for glaucoma. Finally, there are well-documented strains of mice and rats that manifest different types of glaucoma, and because they have a relatively short lifespan, it is possible to study an entire lifecycle of glaucoma onset and progression in an animal analog. Disadvantages include the absence of a lamina cribrosa in the ONH and the small size of the globe, which makes it hard to access clinically. Other anatomical variations in the ONH and trabecular meshwork can limit the use of these models [27,28]. A summary of these studies is presented in Table 1.

#### 3.1.1. Assessment of Genetic and Biochemical Basis of Nicotinamide Supplementation

Several studies have utilized animal models to explore the genetic and biochemical effects of NAM supplementation on neuronal vulnerability in glaucoma. One study investigated [29] the role of the Wallerian degeneration slow allele (*Wld^S^*), a known neuroprotective mutation, in maintaining retinal NAD levels and preventing pre-degenerative changes in glaucoma. DBA/2J (D2) mice, a widely used strain of mice that develops hallmark features of human glaucoma around 9 months of age, were compared to D2 mice with the *Wld^S^* allele (D2 + *Wld^S^*). NAM supplementation was administered to both D2 and D2 + *Wld^S^*, and the combination of *Wld^S^* and NAM supplementation protected a greater proportion of optic nerves with 94% of eyes having no detectable glaucoma (*p* < 0.01 compared to D2 + NAM or D2-*Wld^S^* alone; *p* < 0.001 compared to untreated D2) [29].

In another study [30], low- or high-dose NAM was also given as either prophylaxis or intervention (before or after the onset of high IOP) in D2 and DBA/2J-*Gpnmb+* mice (a variant of D2 mice that do not develop high IOP or glaucomatous change). Notably, high-dose NAM supplementation preserved the optic nerves in 93% of treated eyes (*p* < 0.001) and improved visual function (*p* < 0.01) [30]. Gene therapy with *Nmnat1*, a gene encoding the cellular machinery driving NAD production, was also effective in mitigating ONH and RGC degeneration, and preserving visual function compared to untreated D2 mice [30]. NAM also prevented optic nerve excavation and axon loss in treated D2 mice compared to untreated D2 mice [31]. Genetic findings from 4 to 9 months suggested an age-dependent decrease in critical NAD-producing enzymes Nampt and Nmnat2, and an increase in Nadk, a NAD consuming enzyme. Furthermore, in a comparison of D2 and controls, there was a significant IOP-dependent decrease in Nmnat2. NAM supplementation prevented these transcriptomic changes [31].

These studies have provided evidence for the genetic and biochemical basis for use of NAM in glaucoma. This work has demonstrated NAM’s neuroprotective and positive metabolic effects, allowing for the preservation of optic nerve and RGC structure and function. However, these studies are in animal models, and while we can glean insight from these studies for human trials, we cannot make direct extrapolations between the mouse models and human models. Thus, we need larger randomized control trials with diverse cohorts to be able to test the true genetic and biochemical effects of NAM in humans.

#### 3.1.2. NAM Supplementation Effects on Mitigating Structural Decay of RGCs and Visual Function

NAM supplementation has been found to mitigate structural and functional RGC deterioration in mice with glaucoma, especially during induced oxidative stress. A flicker-pattern electroretinography (F-PERG) study [32] tested the effects of a NAM-rich diet on RGC function. Flickering light exacerbates deficient mitochondrial activity in glaucoma by increasing metabolic demand in RGCs, and F-PERG adaptation (the difference in amplitude between baseline and test F-PERG) measures the ability of RGCs to autoregulate and manage increased metabolic demands induced by flicker. F-PERG adaptation was 41% in 3-month-old D2 mice and diminished with age in D2 more than in NAM-fed D2. At 12 months, F-PERG adaptation was 0% in control mice and 17.5% in D2 mice with nicotinamide-rich diets (*p* = 0.0165). Mice fed with nicotinamide-rich diets also showed ×higher RGC density (2.4×; *p* < 0.01), larger RGC soma size (2×; *p* < 0.01), and a greater intensity of mitochondrial staining (3.75×; *p* < 0.01) [32].

Another study tested [33] the effects of prophylactic systemic administration of NR supplementation in both acute (optic nerve crush) and chronic (induced-ocular hypertension (OHT)) RGC damage mouse models. In the acute model, PERG showed that RGC, inner retinal, and outer retinal photoreceptors were significantly preserved with NR supplementation, compared to control mice [33]. Furthermore, glial fibrillary acidic protein (GFAP) staining showed suppressed retinal inflammation with NR treatment [34]. In the chronic condition, similar findings were observed. NR significantly reduced RGC loss compared to control D2 mice (*p* < 0.0001) [33].

The effects of NR and NAM supplementation have also been shown to differ in either long-term or short-term treatment conditions [34]. On PERG testing, long-term, high dose NR preserved visual function at 9 and 12 months (*p* < 0.001), while long-term, high dose NAM protected visual function loss at 9 months (*p* < 0.001) but not 12 months. High dose NR also protected against RGC loss and optic nerve atrophy, while long-term, high dose NAM treatment significantly protects against optic nerve atrophy only [34].

Dose-dependent neuroprotective effects of NAM have been demonstrated on a wide variety of rat and mouse models representing isolated OHT, axon degenerative (axotomy explant), and mitochondrial degenerative insults (intravitreal rotenone) [35]. In the induced OHT model, the NAM treatment showed no loss of neuroretinal rim and a dose-dependent reduction in RGC loss and nuclear shrinkage compared to normotensive controls [35]. In the axotomy explant model, NAM-treated cultures showed protection against axonal varicosities and preserved dendritic complexity, length, and field area, compared to normotensive controls [35]. Furthermore, NAM treatment provided protection from the effects of rotenone, an inhibitor of complex I of the electron transport chain, maintaining a similar number of RGCs compared to the control mice (*p* = NS) [35].

The studies mentioned above clearly show that various dosing regimens of NAM can protect RGC structure and function in different types of RGC insults, ranging from acute to chronic and toxic to traumatic. However, the data gathered from these studies is gathered in animals and is not easily applied to human glaucoma treatment. The studies vary widely in duration and dosing regimens and use a small number of animal models. It is necessary to run larger randomized clinical trials to better understand the effects of NAM on the structure and function of RGCs in human patients.

#### 3.1.3. Nicotinamide Supplementation and Cytoskeletal Structure

NR supplementation may also confer specific axonal and dendritic protection. In a Wistar rat OHT model, NR treatment prevented retinal axon degeneration and RGC fiber loss in both the center and periphery of the retina (center: *p* = 0.0388 and periphery: *p* = 0.0031) compared to untreated OHT rats [36]. Further cytoskeletal studies [37] examined the effect of NAM supplementation on the RGC microtubule structure and function using second-harmonic generation (SHG) microscopy. The morphology and volume of retinal nerve fibers responded to NAM and exhibited significantly lower age-dependent loss (*p* = 0.049). However, the decrease in the mean density of axonal microtubules was not affected significantly (*p* = 0.43) [37].

A NAM-enriched diet [21] may also provide dose-dependent RGC dendritic protection and preserve the dendritic structure in prophylactic and interventional treatment models in Brown Norway rats. Prophylactic low- and high-dose NAM treatment protected dendritic complexity, dendritic area, and neurite length (all: *p* < 0.0001) compared to untreated OHT rats. Only high-dose NAM showed robust preservation of similar metrics in the interventional condition compared to untreated OHT rats [21].

Early evidence shows that NAM and other NAD precursors can have strong effects on axonal and dendritic morphology and function, including lower age-dependent loss of retinal nerve fiber morphology and volume, and preservation of dendritic complexity and neurite length. However, thus far, there are a limited number of studies examining the ways in which NAM affects axonal and dendritic structure and function in human subjects. These studies also have a limited number of subjects and vary in their protocols and data analysis techniques. Future, large clinical trials of human subjects are necessary to standardize data collection and analysis, and to systematically examine these dynamic systems and how NAM might affect them in human subjects.

### 3.2. Studies of Nicotinamide, Pyruvate, and Glaucoma in Animals and Humans

Clinical research studies involving nicotinamide, pyruvate and glaucoma in animals and human subjects can be divided into three main categories: (1). studies involving NAM, retinal degradation, and vascular parameters, (2). studies involving pyruvate (alone), and (3). Studies involving both nicotinamide and pyruvate combination therapy.

#### 3.2.1. Studies Involving NAM, Retinal Degradation, and Vascular Parameters in Humans and Animals

Pilot works suggest NAM supplementation may improve retinal functionality in human glaucoma subjects. A clinical trial [38] in glaucoma patients was conducted to study the effects of nicotinamide on inner retinal function by measuring photopic negative response (PhNR) and amplitude parameters (Vmax and Vmax ratio) on ERG and perimetry with a visual field (VF) [38]. The Vmax and Vmax ratio improved with nicotinamide supplementation (Vmax, *p* = 0.02; Vmax ratio, *p* = 0.002) compared to the baseline. The difference in the Vmax and Vmax ratio between NAM and placebo also showed a significant increase with NAM supplementation, particularly with a longer duration and higher dosage (Vmax, *p* = 0.03; Vmax ratio, *p* = 0.02). An improvement in the visual field mean deviation (MD) was also observed on NAM and compared to the placebo [38].

A mixed-model clinical trial, including normotensive and OHT Brown Norway rats, postmortem human ocular tissue, glaucoma patients, and healthy controls, was used to investigate NAM and vascular structure [39]. NAM treatment improved mean blood vessel area, percentage area covered by blood vessels, total vessel length, total junctions, junction density, (all *p* < 0.05) and blood vessel wall integrity (*p* < 0.01) compared to untreated OHT rats [39]. Optical coherence tomography angiography (OCTA) data in humans has shown small increases in retinal perfusion density in the ONH and macula (complete image) in healthy controls (0.7%, *p* = 0.04 and 1.0%, *p* = 0.002, respectively) and in the temporal quadrant of the macula in glaucoma patients (0.7%, *p* = 0.02) after NAM treatment [39].

The effect of NAM on retinal function has also been recorded in normal-tension glaucoma (NTG) patients already receiving IOP-lowering therapy [40]. Retinal function was measured using full-field ERG and VF testing. The amplitude change in the photopic negative response in the peak-to-trough (PhNRPT) and the B-wave were significantly greater in the NAM treatment group compared to the placebo (PhNRPT: 3.121 ± 3.968 μV vs. 0.996 ± 4.190 μV, *p* = 0.045; B-wave: 2.112 ± 3.220 μV vs. 0.305 ± 3.279 μV, *p* = 0.032) [40]. This study demonstrates that NAM can enhance electrophysiological retinal function independently of IOP reduction. However, this study is performed in NTG patients and not high-tension POAG. Since NTG is characterized by lower IOP, there may be a slower progression rate, and therefore, a smaller impact on electrophysiological activity of the retina. Further long-term studies in high-tension POAG patients are necessary to elucidate the effect of NAM on electrophysiological function in the retina.

Quality of life (QOL) with regular NAM use has also been measured in POAG patients [41]. Using the Glaucoma Quality of Life-15 Questionnaire, strong evidence showed that NAM supplementation led to significant improvements in QOL scores among glaucoma patients, including central, near, and peripheral vision. Additionally, patients reported improved QOL regarding glare and dark adaptation. NAM also showed significant reduction in IOP in both eyes [41].

Among the studies of NAM performed on humans, one is a study measuring the effects of NAM on ERG and PhNR amplitudes in glaucoma patients and the other is a study of the effects of NAM on the vessel structure of glaucoma patients, measured by OCTA. These studies have small sample sizes and there is difficulty extrapolating these data to larger populations. Furthermore, these studies are pilot in nature and limited in patient diversity, methodological uniformity, and treatment duration. These studies lay the foundation for larger clinical trials to validate these findings.

A summary of nicotinamide interventional studies in human subjects is presented in Table 2. Overall, NAM supplementation has shown an initial degree of efficacy in protecting RGC function and the improvement of retinal vasculature in glaucoma patients.

#### 3.2.2. Studies Involving Pyruvate (Alone) in Humans and Animals

A recent study [17] identified a trend of IOP-dependent decline in retinal pyruvate levels, linked to disturbed glucose metabolism prior to optic nerve deterioration in D2 mice. Oral supplementation of pyruvate in both mouse and induced-OHT SD rat models was strongly protected from neurodegeneration on PERG testing. Visual function improved with pyruvate supplementation in 12-month-old D2 mice compared to untreated 12-month-old mice and did not significantly differ in visual function compared to younger control mice [17]. Pyruvate preserved the RGC axon projections involved in anterograde axoplasmic transport, particularly to the dorsal lateral geniculate nucleus (*p* < 0.01) and superior colliculus (*p* < 0.01) [17]. In the induced-OHT rat model, pyruvate prevented cytoskeletal damage and microglia activation (both *p* < 0.05) compared to untreated OHT rats [17].

In a genetic analysis [43], patients registered in the UK Biobank were examined to assess the impact of plasma metabolites on patients with a high glaucoma polygenic risk score (PRS), and to identify metabolomic signatures of resilience in high-genetic risk individuals. Several elevated glycolysis-related metabolites associated with glaucoma resilience were seen in the top PRS decile, including pyruvate (*p* = 1.9 × 10^−10^). Higher total resilience metabolite levels were associated with lower glaucoma prevalence. Pyruvate was also administered to *Lmx1b^V265D^* mice (a mutation that induces elevated IOP and glaucoma) and prevented anterior chamber deepening compared to untreated mutant mice (*p* < 4.4 × 10^−5^). Pyruvate treatment provided reduction in IOP and mitigation of ONH damage compared to untreated mutant mice [43].

These pilot studies suggest the potential for exploring pyruvate alone or alongside NAM for potentially treating metabolic dysregulation in glaucoma, as evidence suggests that its consumption may slow RGC deterioration, preserve RGC function, and have IOP-lowering capabilities. However, larger studies with sufficient samples and diverse patient populations, linked to long-term vision outcomes in POAG patients, are needed to understand any long-term benefit in POAG care.

#### 3.2.3. Studies Involving Both Nicotinamide and Pyruvate in Human and Animals

Prior studies have identified the possible synergistic benefits that a combination therapy of NAM and pyruvate could have on retinal metabolic function. Both compounds improve glycolytic capacity and increase metabolic efficiency using different mechanisms of action [17,42,43]. A clinical trial was conducted, in which NAM and pyruvate were administered in combination to 32 treated POAG patients over a median period of 2.2 months, showing significant improvement in visual field function [42]. Test locations on VF were higher in the treatment group versus the placebo (median [IQR], 15 [6,7,8,9,10,11,12,13,14,15,16,17,18,19,20,21,22,23,24,25] vs. 7 [6,7,8,9,10,11]; *p* = 0.005). Rates of change in the pattern standard deviation (PSD) also showed improvement with treatment versus placebo (median, −0.06 vs. 0.02 dB per week; 95% CI, 0.02 to 0.24; *p* = 0.02), indicating the improvement of global perimetric sensitivity and rescue of visual function. In contrast, rates of change in MD (0.04 vs. −0.002 dB per week; 95% CI, −0.27 to 0.09; *p* = 0.35) and VFI (0.09 vs. −0.02% per week; 95% CI, −0.53 to 0.36; *p* = 0.71) did not show significant improvement with nicotinamide and pyruvate compared to the placebo [42] (Table 2).

The pilot work on nicotinamide and pyruvate supplementation suggests that the compounds may improve ocular metabolic function and possibly visual function; however, currently, robust data in humans are missing.

### 3.3. Clinical Trials Using Nicotinamide or Nicotinamide + Pyruvate Treatment

There is a current void of properly powered trials establishing proper therapeutic doses, duration, and long-term impact on clinical glaucoma endpoints, including disease status, VF progression, and RNFL thinning. Ongoing clinical trials may help address some of these lingering questions. Among currently registered studies, six new trials are studying the long-term neuroprotective effects of NAM treatment alone [44,45,46,47,48,49]. Other trials are examining the comparative neuroprotective properties of various NAD precursors [50] and the impact of nicotinamide and pyruvate combination therapy [51].

These trials are phase two and three studies that will measure the effects of NAM and similar NAD precursors on several structural and functional outcomes, including VF and OCT, as well as quality of life measures [44,45,46,47,48,49,50,51]. This research is attempting to elucidate the longitudinal effects of these supplements in glaucoma patients and the potential therapeutic benefits in larger, more diverse cohorts. A detailed summary of these clinical trials is presented in Table 3.

## 4. Safety, Side Effects and Adverse Events

While NAM and pyruvate have shown significant neuroprotective benefits in both animal and human subjects, there are potential side effects and prior adverse events that are important to consider when prescribing these supplements in humans. Animal models have shown adverse effects of high-dose NAM, including oxidative DNA damage in hepatic and renal tissue, increased incidence of kidney and pancreatic islet tumors, decreased growth rate or growth inhibition, development of hepatic steatosis and fibrosis, reproductive changes, behavioral deficits, and structural brain changes [52]. NAM use has been associated with side effects such as gastrointestinal discomfort, nausea, bloating, constipation, fatigue, skin rash, vasodilatory effects, and headache [53,54,55]. These side effects are typically transient and do not require serious medical intervention.

NAM is the amide derivative of niacin, and niacin is a known direct hepatotoxic agent, causing changes in serum liver enzyme levels, acute hepatic necrosis, hepatic fibrosis, and jaundice, as discussed in several case reports [53,56,57,58,59]. Drug-induced liver injury (DILI) from niacin consumption is possibly related to intrinsic toxic reaction from overstimulation of nicotinic acid receptors due to high serum levels of niacin, leading to a clinical phenotype that is similar to acute hepatic necrosis [60]. Prior research has shown that adverse events are generally more common with higher doses of oral NAM and niacin, and the tolerability of the dosage depends on comorbid conditions, including renal or liver dysfunction [55]. Furthermore, oral NAM is considered to be safer than niacin, with a lower incidence of adverse events and a more tolerable side effect profile compared to niacin. In some cases, reduction or stopping of the medication was necessary and most side effects were resolved with reduction or stopping of the supplement [55].

Given this association between niacin and DILI, studies utilizing NAM as a therapy for glaucoma have generally excluded participants with a history of liver disease [38,61]. In a prior case report, a graduate student who was prescribed NAM for schizophrenia was found to have recurring bouts of elevated liver enzymes. It was found that he was taking 9 g/day several days before these episodes. Recovery of liver function enzymes was noted when the dosing was reduced to the 2–3 g/day range. This case report demonstrated that doses of NAM (and niacin) well above 3 g/day can be associated with DILI [62]. However, larger studies using NAM treatment showed no evidence of liver dysfunction in an analysis of 244 patients on 3–6 g/day of niacin or nicotinamide [63]. Another study of 55 patients on 1.2 g/day of nicotinamide also showed no evidence of liver dysfunction [64]. In over 1300 participants who have started using NAM in glaucoma clinical trials to date, there have been two reports of severe DILI due to high-dose NAM supplementation [53,65]. The first case took place in a glaucoma clinical trial in the United States and involved a 73-year-old woman with POAG. Upon enrollment in the study, she had normal liver function at the baseline, with no underlying liver disease and no known prescription medications with hepatotoxic effects. After 3 weeks of using NAM at the target dose of 3 g/day, she began vomiting significantly and was found to have elevated liver enzymes. She was hospitalized and diagnosed with DILI. The NAM dose was discontinued, and she was given N-acetylcysteine to treat DILI. Upon discharge, her liver enzymes had normalized, and her condition was stable at follow-up after a year [65]. Another case of DILI occurred in Singapore and involved a 69-year-old Chinese woman enrolled in the Singapore Nicotinamide Trial. Upon enrollment in the study, she had normal liver enzymes and no known risk factors for DILI. She had been taking 3 g/day of NAM when she was found to be icteric and reported significant nausea, vomiting, a rash, and itching. Blood tests revealed significantly elevated liver enzymes. She was told to discontinue NAM and was diagnosed with DILI and terminated from the study. Her symptoms were resolved, and her liver enzymes reached the baseline values within two weeks [53].

DILI often leads to the early termination of involvement in the study for these participants. Less emergent side effects such as rash, nausea, and headaches have also prompted a reduction in dosage when not well-tolerated. In some cases, participant withdrawal from the study is necessary [53]. Therefore, it is essential to conduct larger, randomized clinical trials of NAM and test various dosing regimens to see what level of safety and efficacy is tolerable for the use of NAM in the treatment of glaucoma.

Pyruvate supplementation (either orally or intravenously) is generally well-tolerated in human studies, with mild gastrointestinal symptoms (nausea, diarrhea) being the most common side effects reported. Serious toxic events have not been reported in human supplementation studies [66,67,68]. Thus, pyruvate presents a novel therapeutic avenue for treating mitochondrial dysfunction and oxidative stress in glaucoma, with minimal potential side effects. However, much of the reported data are from case reports of patients on pyruvate therapy regimens. It is necessary to design large-scale, high-power clinical trials to properly test the safety and efficacy of pyruvate supplementation for use in glaucoma treatment protocols.

Importantly, safety remains a significant concern with NAM and pyruvate consumption. The American Glaucoma Society and American Academy of Ophthalmology made several recommendations when treating POAG with NAM: (1). if doses of NAM under 3 g/d are being considered, collaboration with a primary care physician and periodic LFTs are necessary, (2). doses of NAM greater than 3 g/d should be avoided outside of clinical trials where LFTs can be closely monitored, (3) patients should be advised of possible side effects and encouraged to report them immediately, (4) patients with a history of liver disease or current liver disease should not be offered NAM, and (5) Niacin should not be used interchangeably with NAM, as it is known to be hepatotoxic in high doses [53]. It is important to note that current animal studies provide little to no information on safety, and there is still limited information on the long-term safety of these drugs in humans. It is necessary to build a body of literature, systematically assessing the side effects and carefully considering standardized dosing, outcome measures, and adverse effects, and having a surveillance system set up for reporting adverse events [55].

## 5. Current Limitations and Future Areas of Study

To date, studies of the effects of NAM and pyruvate in animal and human models have revealed many important descriptions regarding the role of ROS and oxidative stress linked to glaucomatous disease. Pilot data in animal and human models suggest neuroprotective properties that help preserve RGCs. However, while there are significant data regarding the use of NAM and pyruvate in animal models, data in human POAG patients are scarce and severely limited in scope, patient diversity, methodological uniformity, and treatment duration. Currently, there is a void of longitudinal placebo-controlled studies linking NAM and pyruvate consumption to long-term vision outcomes or structural changes in patients with POAG. There is also a need for studies of pyruvate consumption alone to better understand the genetic, biomolecular, and functional effects of pyruvate monotherapy in human glaucoma patients. While there have been several nicotinamide studies in animals and a handful of human trials which have described the molecular, structural, and functional mechanisms by which nicotinamide may exert a neuroprotective effect in glaucoma, to the best of our knowledge, no study has investigated the effects of pyruvate alone in POAG patients and healthy controls. Several larger registered clinical trials are now underway that may help provide more specificity in terms of findings, although uniformity in methods across studies remains a significant limitation for comparative analysis.

## 6. Conclusions

There is an urgent need to identify novel, modifiable non-IOP metabolic targets for improving POAG care. POAG remains a leading cause of irreversible blindness worldwide, despite a wide array of available therapies targeting elevated IOP. Impaired blood flow and oxygen metabolism have been found in POAG patients. New research suggests that mitochondrial dysfunction may be linked to the increased production of ROS that contributes to RGC death in POAG. NAM and pyruvate support cellular function, show an age-dependent decline and may be lower in POAG patients. In pilot studies, NAM and pyruvate supplementation have resulted in measurable neuroprotective effects and acute improvements in visual function in POAG patients. Comprehensive long-term studies using multimodal imaging in diverse patient populations are needed to establish whether the pilot data and suggestive results to date translate to long-term preservation of vision in POAG patients. Furthermore, full long-term safety profiles for NAM and pyruvate in humans, and at various drug levels, have yet to be established, and they are necessary for incorporating NAM into the gold-standard treatment protocols.

Future clinical trials will need to define safety and tolerability and, more specifically, quantify the effects of NAM and pyruvate on long-term clinical endpoints in POAG patients. Safety, dosing, and efficacy testing of NAM and pyruvate should also be performed in diverse populations to establish risk across age, sex, race and other demographic and socioeconomic considerations. Larger, carefully designed registered trials, including some of those that are currently underway, may help to reveal the potential therapeutic use of NAM, pyruvate, and other neuroprotective agents for POAG.

## Figures and Tables

**Table 1:** Summary of studies on nicotinamide intervention in animal models.

Author	Model	Dose and Duration	Key Findings
Williams et al. [29]	DBA/2J (D2) mice	Dose: 550 mg/kg/d NAM. Duration: 6 months–12 months of age.	The **combination of *Wld^S^* and NAM supplementation protected a greater proportion of optic nerves with 94% of eyes having no detectable glaucoma** (*p* < 0.01 compared to D2 + NAM or D2-*Wld^S^* alone; *p* < 0.001 compared to untreated D2).
Williams et al. [30]	D2 mice	Dose: Low dose (550 mg/kg/d) or high dose (2000 mg/kg/d). Duration: Start at 6 months (prophylactic; prior to IOP elevation) or start at 9 months (intervention; after IOP elevation). Gene therapy with *Nmnat1* injected at 5.5.	Low-dose NAM prevented optic nerve degeneration prophylactically (*p* < 0.01) and as an intervention (*p* < 0.001) compared to untreated D2 controls. **High-dose NAM preserved optic nerves in 93% of treated eyes** (*p* < 0.001) and improved visual function (*p* < 0.01). **Gene therapy with low-dose NAM mitigated ONH degeneration and RGC soma loss** (both *p* < 0.001) and preserved PERG amplitude.
Williams et al. [31]	D2 mice	Dose: Low dose (550 mg/kg/d) or high dose (2000 mg/kg/d). Duration: treated at either 6 or 9 months and examined at 12 months of age).	Genetic findings in control mice from 4 to 9 months suggested an **age-dependent decrease in critical NAD-producing enzymes Nampt and Nmnat2, and an increase in Nadk, a NAD consuming enzyme**. In a comparison of D2 and control mice at 9 months, there was significant IOP-dependent decrease in Nmnat2 in D2 mice compared to controls. **NAM supplementation prevented these transcriptomic changes.**
Chou et al. [32]	D2 mice	Dose: 2000 mg/kg/d NAM vs. no NAM. Duration: 3 to 12 months.	In baseline and test F-PERG, the **normal amplitude decline with age was substantially shallower in the NAM group** compared to untreated D2 (*p* = 0.0135).
Zhang et al. [33]	C57BL/6J mice—ONC model D2 mice—induced OHT model	ONC model: 1000 mg/kg NR, 3×/week for 2 weeks before unilateral ONC, followed by NR treatment the same day and euthanized after 3 or 7 days. Induced-OHT model: 1000 mg/kg NR once the day before and the day of induced-OHT, then NR or PBS 3×/week for 8 weeks.	In ONC model, **RGC, and outer retinal photoreceptor (P1 wave amplitude on PERG), as well as inner retinal (N2 wave amplitude on PERG), functions were preserved in NR**. In induced OHT model, NR significantly reduced RGC loss compared to PBS-treated D2 mice (*p* < 0.0001).
Zhang et al. [34]	D2 mice	NR low dose: 1150 mg/kg/d NR high dose: 4200 mg/kg/d. NAM low dose group: 500 mg/kg/d. NAM high dose: 2000 mg/kg/d.	Long-term, high-dose NR and NAM treatment showed robustly increased retinal NAD levels. Long-term, high dose NR preserved visual function at 9 and 12 months (*p* < 0.001). **Long-term, high-dose NR treatment significantly protected against total RGC loss and optic nerve atrophy** (*p* < 0.00001; *p* < 0.0001, respectively). Long-term, high- and low-dose NR and NAM also prevented iris depigmentation (NAM-low: *p* < 0.01; NR-low: *p* < 0.001; NAM-high and NR-high: *p* < 0.0001).
Tribble et al. [35]	Brown Norway rats—isolated-OHT model C57BL/6J mice—axotomy explant model and rotenone model	Isolated-OHT model—200 mg/kg/d, 400 mg/kg/d, 800 mg/kg/d NAM for 2 weeks. Axotomy explant model— 100 mM or 500 mM NAM for 3 days. Rotenone model—500 mg/kg/d NAM 1 week before rotenone injection.	Induced OHT model: **Dose-dependent reduction in RGC loss** (200 and 400 mg/kg/d: *p* < 0.001; 800 mg/kg/d: *p* = NS) nuclear shrinkage (200 and 400 mg/kg/d: *p* < 0.001, 800 mg/kg/d: *p* < 0.01). Axotomy explant model: NAM treatment protected against RGC loss (100 mM: *p* < 0.05; 500 mM: *p* < 0.01), nuclear shrinkage (100 mM: *p* < 0.001; 500 mM: *p* = NS), axonal varicosities (100 mM and 500 mM: *p* = NS), and **preserved dendritic complexity, length, and field area** (100 mM and 500 mM: *p* = NS). Rotenone model: **Maintained a similar number of RGCs compared to DMSO-treated control mice** (*p* = NS).
Arizono et al. [36]	Wistar Rat—induced OHT model	1000 mg/kg/d NR for 3 weeks (induced OHT group).	NR treatment **prevented retinal axon degeneration and RGC fiber loss in both the center and periphery of the retina** compared to untreated OHT rats. **Oral NR increased p-AMPK levels** (an important neuroprotective molecule) in both OHT and non-OHT groups (OHT + NR: *p* < 0.01; normotensive NR: *p* < 0.05).
Boodram et al. [37]	D2 mice	550 mg/kg/d for 6 months in D2 NAM-fed group.	Second-harmonic generation microscopy showed that **morphology and volume of retinal nerve fibers responded to NAM** and had **reduced age-dependent loss** (*p* = 0.049).
Cimaglia et al. [21]	Brown Norway rats—induced OHT	Dose: 200 mg/kg/d or 600 mg/kg/d. Prophylactic group: start NAM 2 weeks before OHT induction. Intervention group: NAM 3 days after OHT induced.	**Prophylactic low- and high-dose NAM treatment protected dendritic complexity (both: *p* < 0.0001), RGC field area (low dose: *p* < 0.01; high dose: *p* < 0.0001), and dendritic area and neurite length (both: *p* < 0.0001)**. High-dose NAM showed robust preservation of similar properties in the interventional condition.

**Table 2:** Summary of studies on nicotinamide (NAM) intervention or NAM + pyruvate intervention in human glaucoma subjects.

Author	Subjects	Intervention	Key Findings
Hui et al. [38]	*n* = 49 patients completed the study and were included in final analysis	6 weeks of 1.5 g/d NAM, 6 weeks of 3 g/d NAM, followed by crossover without washout	ERG and perimetry with PhNR amplitude parameters showed **Vmax improved by 14.8% (*p* = 0.02) with nicotinamide compared to baseline. Vmax ratio improved by 12.6% (*p* = 0.002) following nicotinamide compared to baseline.** An improvement in visual field mean deviation (MD) was observed with **27% improving ≥ 1 dB on NAM** and only 4% deteriorating compared to placebo (*p* = 0.02). Difference in Vmax and Vmax ratio between NAM and placebo was significantly increased at 12 weeks (Vmax: *p* = 0.03; Vmax ratio: *p* = 0.02).
Gustavsson et al. [39]	*n* = 90 glaucoma patients *n* = 30 healthy controls (also include induced OHT model of Brown Norway rat; postmortem human ocular tissue included)	Animal model: 200, 400, or 800 mg/kg/d over the course of 14 days compared to normotensive and untreated OHT rats. Human model: Received 1.5 g/d NAM for the first week and 3 g/d NAM for the second week.	**AngioTool analysis showed increase in mean vessel area, percentage area covered by vessels, total vessel length, total junctions, junction density (all *p* < 0.05), and vessel wall integrity (*p* < 0.01)** in treated OHT rats in a dose-dependent manner. OCTA data in human subjects showed increases in retinal perfusion density in the ONH and macula complete image in healthy controls (0.7%, *p* = 0.04 and 1.0%, *p* = 0.002, respectively) and **in the temporal quadrant of the macula in glaucoma patients** (0.7%, *p* = 0.02).
Ha et al. [40]	*n* = 53 NTG patients (IOP < 18 mmHg)	NAM or placebo for 12 weeks (NAM 1 g/day for 6 weeks, then 2 g/day for 6 weeks), followed by crossover without washout	**Amplitude changes in the PhNRPT and the B-wave were significantly greater in the NAM group** compared with the placebo group (PhNRPT: *p* = 0.045; B-wave: *p* = 0.032). PhNRPT improved beyond twice the 95% coefficient of variation in 29.0% of the NAM group and 19.3% of the placebo group.
Nicola et al. [41]	*n* = 58 glaucoma patients	Evaluated before and after 6 month period of daily 500 mg oral NAM	**Improvement in QOL scores (mean difference = −2.10, 95% CI: [−2.89, −1.32], *p* < 0.0001), central and near vision (mean difference = −2.16, 95% CI: [−3.91, −0.4], *p* = 0.017), peripheral vision (mean difference = −2.66, 95% CI: [−0.23, −0.08], *p* < 0.001), and the glare and dark adaptation (mean difference = −5.24, 95% CI: [−0.33, −0.14], *p* < 0.001).** Reduction in IOP in both eyes (left eye: mean difference = 0.53, 95% CI: [0.21, 0.86] right eye: mean difference = 0.36, 95% CI: [0.04, 0.68].
De Moraes et al. [42]	*n* = 32 completed the study and included in final analysis	Escalating NAM doses: week 1 1000 mg/kg/d, week 2 2000 mg/kg/d, week 3 to end—3000 mg/kg/d; pyruvate: week 1 1500 mg/kg/d, week 2 to end 3000 mg/kg/d median study period: 2.2 months	**Test locations on VF were higher in the nicotinamide + pyruvate group** versus placebo. Rates of change in pattern standard deviation (PSD) also showed improvement with nicotinamide + pyruvate treatment.

**Table 3:** Summary of clinical trials utilizing nicotinamide, nicotinamide + pyruvate, or nicotinamide + other antioxidant intervention (including supplement type and clinical endpoints).

Trial Name/Number	Location	Trial Design	Primary and Secondary Endpoints
The Glaucoma Nicotinamide Trial (TGNT) NCT05275738 [46]	Sponsor Location: Umeå University, Stockholm, Sweden Other Locations: St Eriks Eye Hospital, Stockholm, Sweden	Prospective, randomized, placebo-controlled, double-masked trial with 2 cohorts: The Swedish Glaucoma Nicotinamide Trial (SGNT) and the Vitamin B3 In Glaucoma Study (VBIGS). SGNT will include healthy and newly diagnosed glaucoma patients. VBIGS will include healthy and diagnosed glaucoma patients on IOP-lowering medications. POAG patients will receive placebo or NAM over 2 years (1.5 g for 6 weeks then 3.0 g onwards).	Primary Endpoints: **Change in VF progression** between the 2 study arms over the 2 year course of the study.
Nicotinamide in Glaucoma (NAMinG): A Randomised, Placebo-controlled, Multi-centre, Phase III Trial (NAMinG) NCT05405868 [45]	Sponsor Location: University College, London, England, United Kingdom Other Locations: 9 other hospitals located throughout the United Kingdom	Phase 3, double-masked, randomized, placebo-controlled trial. Experimental group will receive NAM (1.5 g/day for first 6 weeks, then 3.0 g/day) for up to 27 months, in addition to standard of care IOP-lowering therapy. Control group will receive matching placebo for up to 27 months.	Primary Endpoints: Difference between the treatment arms in **change in VF MD** at 27 months
Efficacy of Nicotinamide on Retinal Ganglion Cell Functions in Glaucoma Patients NCT06078605 [44]	Sponsor Location: CHA University Bundang Medical Center, Seongnam, Bundang-gu, Republic of Korea	Clinical trial, cross-over design, double-blind, placebo-controlled, randomized, single-center study. NAM Group Baseline—6 weeks: (1.0 g/day, QD) 6–12 weeks: (2.0 g/day, BID) Crossover 12–18 weeks: (1.0 g/day, QD) 18–24 week: (2.0 g/day, BID) Placebo Group Baseline—6 weeks: (1.0 g/day, QD) 6–12 weeks: (2.0 g/day, BID) Crossover 12–18 weeks: (1.0 g/day, QD) 18–24 weeks: (2.0 g/day, BID).	Primary Endpoints: Measuring the effects of nicotinamide on RGC function by **measuring PhNR amplitude on ERG intra-group at baseline and 12 weeks**.
Nicotinamide Supplementation in Glaucoma (TAMING) NCT06731582 [47]	Sponsor Location: Singapore Eye Research Institute, Central Singapore, Singapore Other Locations: National University Hospital, Central Singapore, Singapore	Phase 3, randomized, placebo-controlled, double-masked, superiority trial with two parallel groups. The period of supplementation for both the treatment and placebo group will be 24 months. NAM group will take a dose of 1.5 g/day (2 tablets of 750 mg each). Placebo group will take a dose of 2 tablets/day.	Primary Endpoint: **≥30% reduction in VF MD progression measured over 2 years** compared to current IOP-lowering treatments alone. Treatment duration will be 2 years.
Nicotinamide and Pyruvate for Open Angle Glaucoma: A Randomized Clinical Study NCT05695027 [51]	Sponsor Location: Columbia University Irving Medical Center, New York City, New York, United States Other Locations: 2 other hospitals in the United States (one in New York and one in California)	Phase 2/3, randomized, placebo-controlled, single-blind, with 2 parallel groups. Patients will continue taking IOP-lowering glaucoma medication. Experimental group will receive N&P. The N&P group will receive N&P for 87 weeks (20 months). Control group will receive placebo for 87 weeks (20 months).	Primary Endpoint: **Change in visual field results based on pointwise and global metrics** between intervention and placebo groups over 87 weeks. **Changes in retinal nerve fiber and ganglion cell layer thickness** as assessed by optical coherence tomography (OCT) between groups.
Effect of Niacinamide Supplementation in Glaucoma (NiSPoG) NCT07007260 [48]	Sponsor Location: University of Medicine and Pharmacy Craiova, Craiova, Romania Other Locations: 2 other hospital locations in Romania	100 adult subjects with POAG will take NAM (500 mg) every day for 1 year. The patients will visit the clinic at 3, 6, and 12 months for testing.	Primary Endpoint: Logarithm of the Minimum Angle of Resolution (logMAR; represents visual acuity as a logarithmic value, i.e., ETDRS chart) **visual field parameters.** Peripapillary retinal nerve fiber layer thickness (pRNFL). Visually evoked potential latency of P2 wave when using a flash stimulus. Glaucoma QOL-15 questionnaire.
Nicotinamide Levels in Serum, Aqueous Humor, and Tear Film in Glaucoma and Correlations with Mitochondrial Damage-Associated Molecular Patterns (mtDAMPs) and Senescence-Associated Secretory Phenotype (SASP) NCT07006194 [49]	Sponsor Location: University of G. d’Annunzio Chieti-Pescara, Chieti Scalo, Chieti, Italy Other Locations: 2 other hospital locations in Italy	Cross-sectional, case–control, factorial assignment, multi-center study measuring NAM levels in ocular and non-ocular fluids at different stages of glaucoma and correlate with disease severity and mitochondrial function. Four patient groups: (1) Uncontrolled glaucoma patients scheduled for glaucoma surgery. (2) Controlled glaucoma patients scheduled for cataract surgery. (3) Healthy controls undergoing cataract surgery. (4) Pharmacologically controlled glaucoma patients supplemented with NR.	Primary Endpoints: **(1) NAM levels in serum, aqueous humor, tear film.** **(2) Correlate NR concentrations in biological fluids with disease stage measured by MD, VFI, and Hodapp classification.** (3) Correlate NAD concentrations with mitochondrial function markers (mtDAMPs). (4) NAD levels in PBMCs from controlled POAG patients after 4 weeks of NR supplementation.
Comparisons of NAD Precursors for Neuroenhancement in Glaucoma Patients NCT06991712 [50]	Sponsor Location: Hong Kong University Eye Centre, Wong Chuk Hang, Hong Kong	Phase 2, randomized, clinical trial with parallel assignment in 6 total groups: 4 groups receiving equimolar NR (300 mg/d), NAM (125 mg/d), nicotinic acid (NA) (125 mg/d), and nicotinamide mononucleotide (NMN) (350 mg/d) 2 groups taking placebo Phase 1—randomized to placebo, NR, or NAM Phase 2—randomized to placebo, NA, or NMN	Primary Endpoints: **Changes in VF sensitivity using HVF 24-2 standard VF** (VFI and dB changes) from baseline and after 2 weeks of supplementation
